# The First Fossil Record of the Genus *Manica* Jurine, 1807 from Late Eocene Baltic Amber and Discussion of the Early Evolution of Myrmicini (Hymenoptera: Formicidae: Myrmicinae)

**DOI:** 10.3390/insects14010021

**Published:** 2022-12-24

**Authors:** Dmitry Zharkov, Dmitry Dubovikoff, Evgeny Abakumov

**Affiliations:** Department of Applied Ecology, Faculty of Biology, Saint Petersburg State University, 199034 Saint Petersburg, Russia

**Keywords:** Formicidae, fossil ants, *Manica*, Eocene Baltic amber, micro-computed tomography (µCT), 3D model

## Abstract

**Simple Summary:**

In this study, a new fossil ant species from the Baltic amber, †*Manica andrannae* Zharkov and Dubovikoff, sp. n., is described based on the worker. The main morphological features are studied and illustrated using X-ray micro-computed tomography (µCT), including µCT-based palaeoreconstruction (3D modelling). The newly described taxon is the first fossil species of the genus *Manica* Jurine, 1807 and represents an important link in understanding the origin and evolution of the genus. Despite the finding of a first fossil species in the Old World, based on the study of a new species and comparing it with other members of the genus, we assume the origin of the genus *Manica* was North America during the early Eocene (about 50 Ma) and the later distribution of its representatives in Eurasia occurred in the middle of the Eocene or earlier. Relationships with other species of the genus and within tribe Myrmicini are discussed.

**Abstract:**

The Holarctic genus *Manica* Jurine, 1807 are mysterious and primitive ants from the tribe Myrmicini of the subfamily Myrmicinae. The first fossil species of this genus, †*Manica andrannae* Zharkov and Dubovikoff, sp. n. is described from the Baltic amber (ca. 33.9–37.8 million years ago). X-ray microcomputed tomography (µCT) was used to access morphological features and accurately measure the new species. A straightened and painted 3D model is also proposed as a reconstruction of the worker. The new species differs from all extant species of the genus by the propodeum with a weakly convex dorsum and short, blunt tubercles, and by more angular petiolar node. These features bring it closer to sister genus *Myrmica* Latreille, 1804. The phylogenetic relationships of the new species with other species of the genus are discussed. Based on the studied morphological features, the species is closest to the species *Manica yessensis* Azuma, 1955. The early evolution and paleobiogeography of the tribe Myrmicini are discussed. This finding confirms the origin of the genus *Manica* at least in the Eocene epoch.

## 1. Introduction

The genus *Manica* Jurine, 1807 belongs to the tribe Myrmicini of the largest ant subfamily Myrmicinae, but it includes only six valid described species four of which occur in the Nearctic and two in the Palearctic: one in Europe and one in Japan [1]. They are primarily montane ant species: *M. rubida* (Latreille, 1802) distributed in Europe, Asia Minor, and the Caucasus; *M. bradleyi* (Wheeler, 1909), *M. hunteri* (Wheeler, 1914), *M. invidia* (Bolton, 1995), and *M. parasitica* (Creighton, 1934) occur in North America west of the hundredth meridian and north of 33° N; *M. yessensis* Azuma, 1955 in the north and center of Japan. Colonies are small, nests are usually found in open habitats with low vegetation, commonly under stones or in underground chambers and galleries with vertical entrances in the form of craters [2,3,4]. The biology and ethology of *Manica* ants are little known, in general they are similar to species of the sister genus *Myrmica*, but they are more primitive [4]. One of the North American species (*M. parasitica*) is a social parasite (an inquiline) [5]. Here, we describe a new fossil ant species from late Eocene Baltic amber, †*Manica andrannae* sp. n., which represents the first fossil record of this genus, significantly complementing and expanding our knowledge on the Eocene diversity of this family. Previous authors have proposed that the genus *Manica* originated in western North America [2,6,7], and our new find does not contradict these assumptions (see Discussion below).

## 2. Materials and Methods

**Figure 1:**
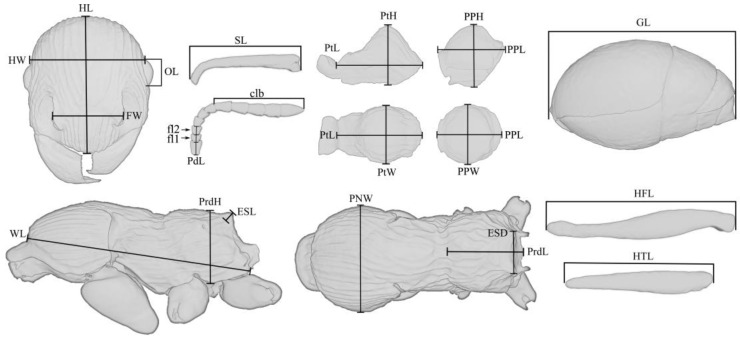
Main measurements of the †*Manica andrannae* sp. nov. (explanation in the text above).

**Figure 2:**
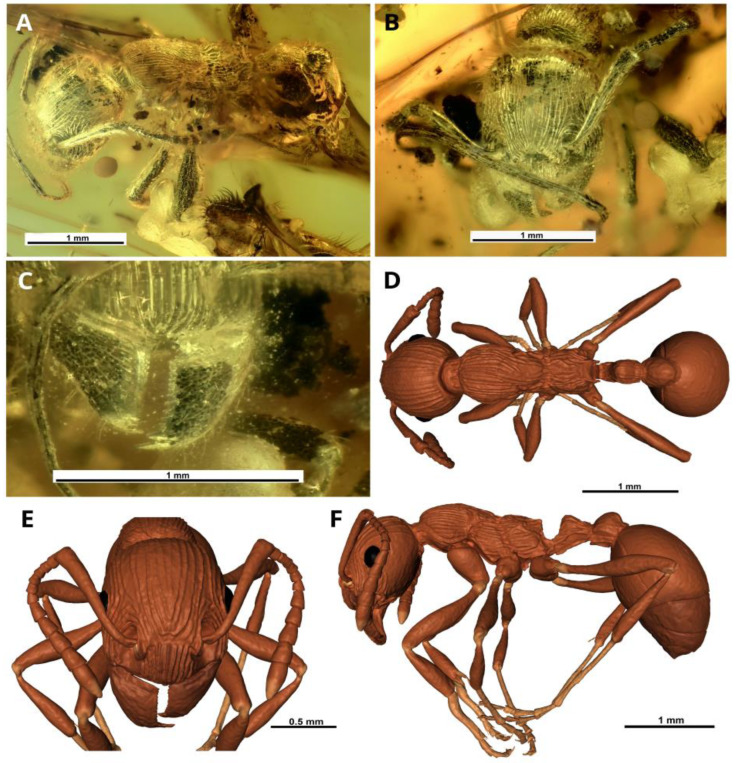
†*Manica andrannae* sp. nov., holotype, photomicrographs (**A**–**C**) and paleontological reconstruction (3D models) (**D**–**F**). Worker: **A**,**F**—habitus, left lateral view; **B**,**E**—head, frontal view; **C**—clypeal margin and mandibles; **D**—habitus, dorsal view.

**Figure 3:**
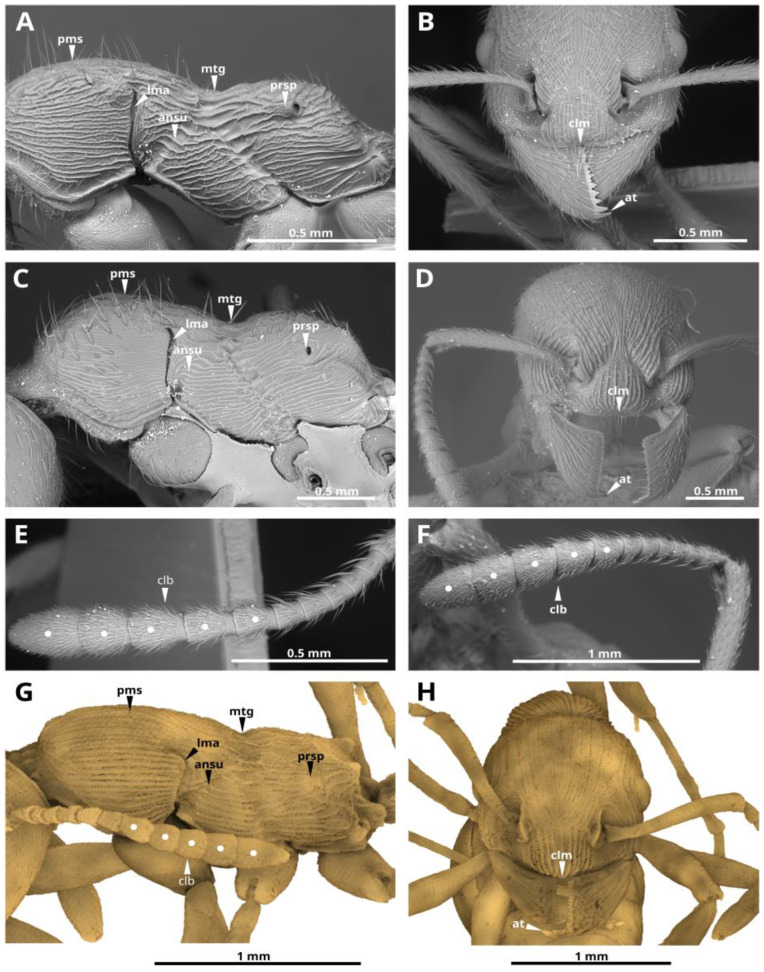
Select key features of the *Manica* species. SEM micrographs of *M. invidia* (**A**,**B**,**E**), *M. rubida* (**C**,**D**,**F**) and µCT volume rendering of †*M. andrannae* sp. n. (**G**,**H**). Worker: **A**,**C**,**G**—mesosoma, left lateral view; **B**,**D**,**H**—head, frontal view; **E**,**F**—flagellum. Abbreviations: promesonotal suture (**pms**), metanotal groove (**mtg**), propodeum spiracle (**prsp**), lateropronotal mesopleural articulation (**lma**), anapleural sulcus (**ansu**), clypeal margin (**clm**), apical tooth (**at**), antennal club (**clb**).

The studied specimen of fossil ant (Figure 1, Figure 2 and Figure 3G,H; Supplementary Materials) originated from the Baltic amber, Priabonian age (Late Eocene, 37.8–33.9 Ma) [8], the Prussian Formation, on the Sambia Peninsula near Kaliningrad, Kaliningrad region, Russia. The holotype of the new species is kept in the collection of the Kaliningrad Amber Museum, Kaliningrad, Russia, under the collection number KAM BX 34/22.1. The amber was hand processed for study (cut and polished) by one of the authors (DD) at the St. Petersburg State University (PalaeoEntLab).

The studies were performed on the equipment of the Research Park of St. Petersburg State University (“Centre for X-ray Diffraction Studies”, project No. 103-23769; “Resource Centre for Microscopy and Microanalysis”, project No. 112-23465 and “Computing Centre”, project No. 110-27449).

Photography and morphological analysis of samples were performed using a Leica M205C motorized stereomicroscope. SEM micrographs were obtained on Desktop Scanning Electron Microscope Hitachi TM3000 (Hitachi Corp., Tokyo, Japan). Subsequent image processing was carried out using the Helicon Focus Pro 8, Kritta 5.0.2 and Inkscape 1.2 software. 

Micro-computed tomography allowed us to make accurate measurements of the specimen and to study all of the traditional diagnostic features of the new species. For a clearer graphical representation of the information, we made a reconstruction of a new species in the form of a digitally painted and straightened 3D model. Arrays of microtomographic sections were obtained using a desktop high-resolution X-ray microtomograph SkyScan 1172. Visualization, volume rendering, and segmentation of tomographic sections were performed in 3DSlicer 5.1 and Drishti 3.0 software. The holotype was scanned with the following parameters: voltage 40 kV, current 250 µA, without a filter, with a pixel size of 2.46 microns and a resolution of 2848 × 2692 pixels per slice with a continuous 360° rotation, and a shutter speed of 1300 ms per frame (2268 X-ray projections).

The inclusion was inverted and subsequently isolated from the surrounding background using the software segmentation editor. Part of poorly preserved morphological structures were previously segmented in the segmentation editor by manually labeling each slice.

The measurements indicated below were performed by volume rendering of the sample in 3DSlicer which made all morphological structures available for study (as opposed to studying samples directly in amber with the help of a microscope) and made it possible to measure with precision of 0.01 mm. The results of segmentation (in 3DSlicer) in the file format PLY were imported into the free and open Blender 3.1 software for “straightening” (changing the position of limbs and other parts of the body), painting, and animation of the ant reconstruction. The sample was “straightened” using the Auto-Rig PRO tool. All the individual parts of the reconstruction were transformed into polygonal grids using the Smart UV project tool and painted manually (the color of the models is not the natural color of the specimen or the assumed lifetime color).

The dimensional values of morphological structures are given in millimeters. The following designations are used in the text:

**HW**—head width, measured along the upper line of the eyes;

**HL**—head length, maximum length of the head, measured from the posterior margin of the head to the anterior-most extremity of the clypeus;

**SL**—the maximum straight-line length of the scape measured from antennal bulb to the apex;

**FW**—the maximum width measured between the frontal lobes;

**PdL**—pedicellum length;

**FI_1_**—the length of the first flagellomere;

**FI_2_**—the length of the second flagellomere;

**OL**—the maximum eye length measured by maximum diameter;

**MdL**—the length of the mandible, measured from the mandibular apex to the anterior clypeus margin, or to the transverse line connecting the anterior-most points in those taxa where the margin is concave medially;

**WL**—Weber’s length, the diagonal length of the mesosoma in profile from the point at which the pronotum meets the cervical shield to the posterior basal angle of the metapleuron;

**PNW**—the maximum width of the pronotum in dorsal view;

**ESL**—maximum length of propodeal tubercles (or spines) in profile, measured along the tubercle/spine from its tip to the deepest point of the propodeal constriction at its base;

**ESD**—distance between the tips of propodeal tubercles/spines in dorsal view;

**PtL**—the length of the petiolar node in profile, measured as the distance from the place of attachment to the propodeum to the place of attachment to the postpetiole;

**PtH**—the height of the petiolar node in profile, measured as the perpendicular distance from the ventral edge to the highest point of the petiolar node;

**PtW**—the maximum width of the petiolar node in dorsal view;

**PPL**—the length of the postpetiole in profile, measured as the distance from the place of attachment to the petiolar node to the place of attachment to the gaster;

**PPH**—the height of the postpetiole in profile, measured as the perpendicular distance from the ventral edge to the highest point of the postpetiole;

**PPW**—the maximum width of the postpetiole in dorsal view;

**HFL**—the maximum length of hind femur, measured in anterior view;

**HTL**—the maximum length of hind tibia, measured in anterior view;

**PrdL**—the maximum length of the propodeum in dorsal view;

**PrdH**—the height of the propodeum in profile, measured as the perpendicular distance from the ventral edge to the highest point of the propodeum;

**GL**—the length of the gaster, measured as the distance from the place of attachment of the postpetiole to the top of the gaster in ventral view;

**TL**—the total length of the ant (=HL + MdL + WL + PtL + PPL + GL).

Indices:

**CI** (cephalic index) = **HL/HW**

**SI_1_** (scape length index) = **SL/HL**

**SI_2_** (scape width index) = **SL/HW**

**FLI** (frontal lobes index) = **FW/HW**

**OI_1_** (eye length index) = **OL/HL**

**OI_2_** (eye width index) = **OL/HW**

**PI_1_** (petiole height index) = **PtL/PtH**

**PI_2_** (petiole width index) = **PtL/PtW**

**PPI_1_** (postpetiole height index) = **PPL/PPH**

**PPI_2_** (postpetiole width index) = **PPH/PPW**

**PPI_3_** (postpetiole-petiole index) = **PPW/PtW**

**ESLI** (propodeal spine length index) = **ESL/HW**

**ESDI** (propodeal spine distance index) = **ESD/ESL**

**MI** (mesosomal index) = **WL/PNW**

**PRI** (propodeal index) = **PrdL/PrdH**

## 3. Results

### 3.1. Systematic Palaeontology

**Family Formicidae Latreille, 1809**

**Subfamily Myrmicinae Lepeletier de Saint-Fargeau, 1835**

**Genus *Manica* Jurine, 1807**

†***Manica andrannae*** Zharkov and Dubovikoff, sp. n.

(Figure 2 and Figure 3G,H; Supplementary Materials)

**Type material**. Holotype: worker, KAM BX 34/22.1.

**Type stratum.** Late Eocene, Priabonian age (37.8–33.9 Mya).

**Type locality.** Russia: Kaliningrad Region, Baltic Sea coast, Sambia (Samland) Peninsula, Yantarny (formerly Palmnicken).

**Etymology.** The name is derived from parts of the names of our colleagues and friends Andranik Manukyan and Anna Smirnova (Kaliningrad Amber Museum).

**Description.** Worker. The body length (TL) is 5.96 mm. Head longer than broad, the occipital angles broadly rounded. Eyes oval, moderately convex, set at the middle of the side of the head. Frontal carinae short, bluntly pointed in front, merge with the rugae, which surround antennal sockets. Clypeus is prominent, anterior clypeal margin very broadly rounded. Masticatory margin of mandibles with two large apical and preapical teeth followed by 14 min teeth or denticles. Palps are short, with 6 maxillary and 4 labial palpomeres. Antennal scapes reaching posterior border of the head; abruptly bent at the base. Second flagellar joint is broader than long, and the third joint is longer than broad, joints 2–6 gradually increase in diameter and length, the five apical joints together form a distinct club (Figure 2A,E,F and Figure 3G; Supplementary Materials). Mesosoma in profile with the promesonotum rather feebly convex, the promesonotal suture not impressed, but distinctly visible. Lateropronotal mesopleural articulations are well defined. Metanotal groove deeply impressed, both dorsally and laterally. Propodeal dorsum with two posterolateral tubercles that are short, straight, wide, blunt at the apex, directed upward and slightly backward (Figure 2A,F and Figure 3G; Supplementary Materials). The propodeal spiracles are strongly bulging, directed backwards (Figure 2F and Figure 3G; Supplementary Materials). Metasternal process a pair of well-defined convex thickened lobes with visible ventral midline between them. Petiole with angular node, petiolar sternite anteriorly with a longitudinal, keel-shaped process. Postpetiole with a prominent, ventral process, broad from the bottom and acute from the side view (Figure 2F; Supplementary Materials). Clypeus, frontal area, dorsal faces of head, mesosoma and petiole longitudinally rugose (Figure 1). A simple (but moderately wide) spur is present on meso- and metatibiae. Possibly they are pectinate, but that is unclear on volume rendering and invisible on specimen in amber. Gaster relatively bulky, oval. Standing hairs abundant on whole body. Extensor surface of the femur, tibia, tarsus, antennal scapes and the proximal half of the flagella covered with small, fine decumbent hairs. Club of the flagellum pubescent.

Males and queens are unknown.

Measurements (mm). HL 1.26; HW 0.99; FW 0.5; SL 1.01; PdL 0.16; FI1 0.08; FI2 0.09; OL 0.26; MdL 0.55; WL 1.67; PNW 0.73; ESL 0.07; ESD 0.26; PtL 0.56; PtH 0.39; PtW 0.31; PPL 0.34; PPH 0.46; PPW 0.39; HFL 1.42; HTL 0.98; PrdL 0.48; PrdH 0.58; GL 1.58; TL 5.96.

**Indices.** CI 1.27; SI1 0.8; SI2 1.02; FLI 0.5; OI1 0.21; OI2 0.26; PI1 1.42; PI2 1.82; PPI3 0.31; PPI1 0.73; PPI2 1.18; PPI3 1.29; PPI4 0.4; ESLI 0.07; ESDI 3.88; MI 2.29; PRI 0.83.

### 3.2. Comparison

†*M. andrannae* sp. n. shares a typical habitus with extant *Manica* species and can be undoubtedly placed within the genus based on the combination of the following characters: antennal club 5-segmented, promesonotal suture faint but visible on dorsum, lateropronotal mesopleural sutures are well developed, metonotal groove strongly impressed, metasternal process a pair of well-defined convex thickened lobes with visible ventral midline between them, mandibles with a prominent apical tooth and a prominent subapical tooth, the remaining 12–16 teeth minute, the rugae occur in parallel lines on the thorax, palps are short, maxillary of 6 segments, labial of 4. The propodeal spiracles are convex, directed backwards. From all recent species of the genus, the new species is distinguished by the more angular petiolar node (such as some *Myrmica* Latreille, 1804*,* while evenly rounded in recent species of *Manica*), the dorsal surface of the propodeum is less convex with the distinct tubercles on the propodeum, which is clearly a plesiomorphic condition, which, apparently, disappeared over time in recent representatives of the genus.

## 4. Discussion

Over the past 65 million years, geological events and changing climatic conditions have profoundly affected the distribution of plants and animals [9]. Biogeographic data and paleontological findings may provide some clues about the diversification of the tribe Myrmicini and the origin of its modern genera. Based on the estimates of Ward et al. [7], the Myrmicini tribe forms a monophyletic group that is sister to all other in the Myrmicinae subfamily. Myrmicini is considered morphologically the most primitive in the subfamily Myrmicinae, based on the plesiomorphic states of many features such as 6−segmented maxillary and 4−segmented labial palps, 11–12−segmented antennae in females, and 12–13−segmented antennae in males, usually a well-developed pectinate spur on the middle and hind tibiae, the mesosomal structure with clearly defined sutures (what is noted in the genus *Manica*, for example see Figure 2 and Figure 3; Supplementary Materials), etc., [6]. Moreover, in comparison with *Myrmica*, the genus *Manica* has a more “primitive” biology [10,11,12]. Given the plesiomorphic features, the modern subordinate position of *Manica* species in modern montane ant societies and absence of paleontological finds (prior to this work), there is limited reason to think that *Manica* were once widespread ants. This may be an advantage due to the lower prevalence of dominant species of ants in such an environment. Representatives of the genus *Manica*, as in the case of its related genus *Myrmica* and some other representatives of the subfamily Myrmicinae, are among the ants subordinate in the hierarchy of dominance and, therefore, less able to protect resources [13]. Considering the modern lifestyle of *Manica* i.e., confined to the mountains, with small colonies, and with workers rarely coming to the surface [2], the finding of this genus in amber was rather unlikely.

**Figure 4:**
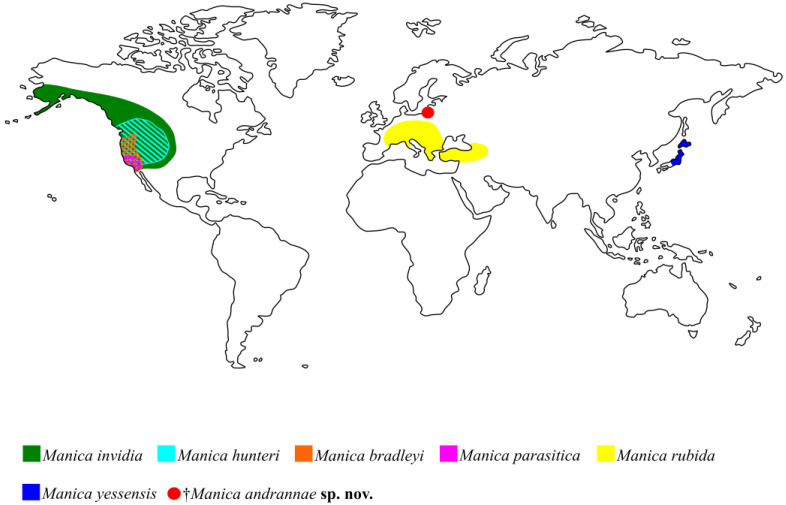
Diagrammatic representation of the distribution of the *Manica* species: filled and shaded area—recent species; red circle—fossil record from Baltic amber.

Presently *Myrmica* species are distributed mainly in the Holarctic, but also in the northern parts of the Indomalayan and Neotropical regions. *Manica* has an exclusively Holarctic distribution (Figure 4). There is not much paleontological evidence for the geographical origin of the genus *Myrmica*. In comparison with numerous other recent genera, the samples of *Myrmica* fossils are quite rare and occur exclusively in late Eocene European amber [14]. Even considering that Eocene European amber is the most studied fossil ant fauna in the world [15,16,17], only five species of *Myrmica* [14,18,19], two species of *Plesiomyrmex* Dlussky and Radchenko, 2009 and *Protomyrmica* Dlussky and Radchenko, 2009 [20], and one species of *Manica* described here are known for the Myrmicini tribe. Their lifestyle hardly favored getting them into the resin, but by the late Eocene they, apparently, were already quite diverse.

Recent *Myrmica* species in Central and Southeast Asia (Himalayas, southern China, Burma, Thailand, Vietnam, and Taiwan) demonstrate the most diverse morphology and also contain the largest number of species, most of which are endemic and have many plesiomorphic states. For example, *Myrmica mirabilis* Elmes and Radchenko, 1998, has primitive features that can bring it closer to *Manica*: general shape and large body size, promesonotal suture weak in dorsal view, non-obvious 4-jointed apical club (it happens to *Manica* too) [21]. Multidentate mandibles with more than 10 denticles of *Myrmica hecate* Weber, 1947 is also a feature characteristic of the genus *Manica*. In more southern areas, species of *Myrmica* live exclusively in mountain forests and alpine meadows at altitudes of more than 1200–2000 m, and sometimes even higher than 4800 m [6,14]. For example, the west Palearctic ant paleofauna was very similar in composition (at generic level) and structure to the ones observed now only in the mountain area of the Indomalayan region [22]. That is, they are being displaced, such as *Manica*, by more competitive species. It seems logical to assume that the genus *Myrmica* first appeared in the World, and not in the New World, where none of the species is similar to the species found in Late Eocene amber, unlike the species of the Old World. Paleontological data from amber show that the species of the *ritae* group and other ancient forms of *Myrmica* were certainly present in Europe in the Late Eocene (ca. 37.8–33.9 Mya). *Manica*, in turn, has 4 out of 6 species that live in North America (Figure 4) [1]. Based on the morphology of *Manica* and taking into account our new finding, we can suggest the place of origin of this genus. Wheeler and Wheeler [2] state that *Manica* originated from the New World based on the presence of a larger number of species in the Nearctic and what is found there, according to the authors, the putative ancestral species—*M. invidia*. They considered *M. invidia* as the supposed ancestor, based on the fact that this species is the most “plastic” and has the largest range. However, the authors assumed the origin of *M. rubida* in the Old World, not yet knowing about the existence of *M. yessensis* in Japan. Radchenko and Elmes [23] also believed that place of origin of *Manica* is most likely the New World.

Three of the six extant species, namely the Nearctic *M. hunteri* and the Palaearctic *M. rubida* and *M. yessensis* have a ventral postpetiolar process, similarly to the new fossil *†M. andrannae* sp. n.. *M. hunteri* also has the anterior clypeal margin medially unnotched, such as the fossil species. Based on these morphological features, it can be assumed that the ancestral state observed in *M. hunteri*, *M. invidia,* and *M. rubida* are very similar, but *M. invidia* differs from these species in the absence of a ventral process of postpetiole and the presence of deep medially notched clypeus. †*M. andrannae* sp. n. is most similar to *M. yessensis* in general body rugosity, as well as *M. yessensis* has a sharp edge of the propodeum, as if there are small teeth. It seems unlikely that the ancestor of *Manica* spread from Europe to Asia and vice versa, even through the existing shores or islands of Tethys Ocean. Since, apparently, the Western Palearctic fauna developed in a different way to a greater extent isolated from the Eastern Palearctic fauna [9]. This suggests that *Manica* could have formed in the New World, after which the ancestor of *M. yessensis* got to the eastern Palearctic through Beringia, and the ancestor of *M. rubida* to Europe through Thulean Bridge. 

Based on the discussion above, we assume that the center of origin of *Manica* was in the New World. If this scenario is accepted, then the common ancestor of the modern genera Myrmicini must have existed before the complete separation of North America and Eurasia. From the Mid Cretaceous (ca. 100 Mya) throughout the Tertiary, northeastern Eurasia was almost permanently connected to the Nearctic via Beringia [24,25,26]. Additionally, the Atlantic Thulean and De Geer routes connected Europe and eastern North America in the late Cretaceous and Early Tertiary [26,27]. Most important for the exchange of flora and fauna was the Thulean Bridge, which connected Europe to Greenland via the British Isles and persisted until the early Eocene (about 50 Ma) [9,23,25]. On this basis, it could be assumed that the ancestor of Myrmicini had already evolved by the time of the destruction of the Thulean Bridge (50 Mya), which corresponds to the estimated time of origin of tribe based on molecular and paleontological data [7].

Interesting in the context of this work is †*Myrmica paradoxa* Radchenko et al., 2007 [14] from Bitterfeld amber, which looks superficially similar to †*Manica andrannae* sp. n. in some features: it has short propodeal tubercles that are not found in other *Myrmica*, and the promesonotal suture is faint but visible on the dorsum. However, according to the set of features in the description, and which can be seen from the only and poor photo, it is unlikely to be a *Manica* (3 jointed club, uncharacteristic head sculpture for *Manica*). Micro-computed tomography scanning is required to check all available morphological features in more detail of †*Myrmica paradoxa*.

Our finding does not contradict, but rather confirms the idea of Radchenko and Elmes [23], that *Manica* and *Myrmica* most likely originated from a common ancestor at about the same time about 50 million years ago during the surge of diversification of ants and before North America was completely separated from Eurasia. The common ancestor of these genera apparently had spines on the propodeum, a full complement of mesosomal sutures and the promesonotal suture impressed in dorsal view. However, if we take into account the modern diversity of these genera and the paleontological finds, it seems that these genera diverged much earlier than in the Eocene. *Manica* could have formed in the New World and then ended up in the Palearctic, and *Myrmica* could have formed in the Old World.

## Data Availability

All the required data relevant to the presented study are included in the manuscript.

## Supplementary Materials

The following supporting information can be downloaded at: https://www.mdpi.com/article/10.3390/insects14010021/s1, Video S1: †*Manica andrannae* Zharkov & Dubovikoff, sp.nov., holotype, worker, palaeontological reconstruction (3D model, video); File S1: †*Manica andrannae* Zharkov & Dubovikoff, sp.nov., holotype, worker, palaeontological reconstruction (3D model, .stl file).
